# Supplementary material for: Nicotinamide adenine dinucleotides are associated with distinct redox control of germination in Acer seeds with contrasting physiology
Source: PLoS One. 2021 Jan 27;16(1):e0245635. doi: 10.1371/journal.pone.0245635 (PMC7840005; doi:10.1371/journal.pone.0245635)
Supplement: S1 Fig — Correlation matrices calculated for embryonic axes (A) and cotyledons (B) of Norway maple, and embryonic axes (C) and cotyledons (D) of sycamore between concentrations of nicotinamide dinucleotide (NAD) phosphate (NADP) redox couples and their ratios, levels of ascorbate (Asc), ascorbic acid (AsA), dehydroascorbate (DHA), reduced (GSH) and oxidized (GSSG) glutathione, catabolic redox charge (CRC), anabolic redox charge (ARC), dormancy depth, phosphorylation capacity of isoform 1 (NADK1) and isoform 3 (NADK3) of NAD kinase, reducing power and half-cell reduction potential of glutathione (EGSSG/2GSH) and ascorbate (EDHA/AsA). Proportional data were transformed prior to analysis using the arcsine transformation. Crossed numbers indicate non-significant correlation (P > 0.05). (DOCX) [file pone.0245635.s001.docx]

**S1 Fig. Correlation matrices**. Correlation matrices calculated for embryonic axes (A) and cotyledons (B) of Norway maple, and embryonic axes (C) and cotyledons (D) of sycamore dry and germinating seeds between concentrations of nicotinamide adenine dinucleotide (NAD) and its phosphate (NADP) redox couples (reduced NAD(P)H and oxidized NAD(P)+) and their ratios, levels of ascorbate (Asc), ascorbic acid (AsA), dehydroascorbate (DHA), reduced (GSH) and oxidized (GSSG) glutathione, catabolic redox charge (CRC), anabolic redox charge (ARC), dormancy depth, phosphorylation capacity of isoform 1 (NADK1) and isoform 3 (NADK3) of NAD kinase, reducing power and half-cell reduction potential of glutathione (*E*_GSSG/2GSH_) and ascorbate (*E*_DHA/AsA_). Proportional data were transformed prior to analysis using the arcsine transformation. Crossed numbers indicate non-significant correlation (P > 0.05).

**A)** Norway maple embryonic axes

**
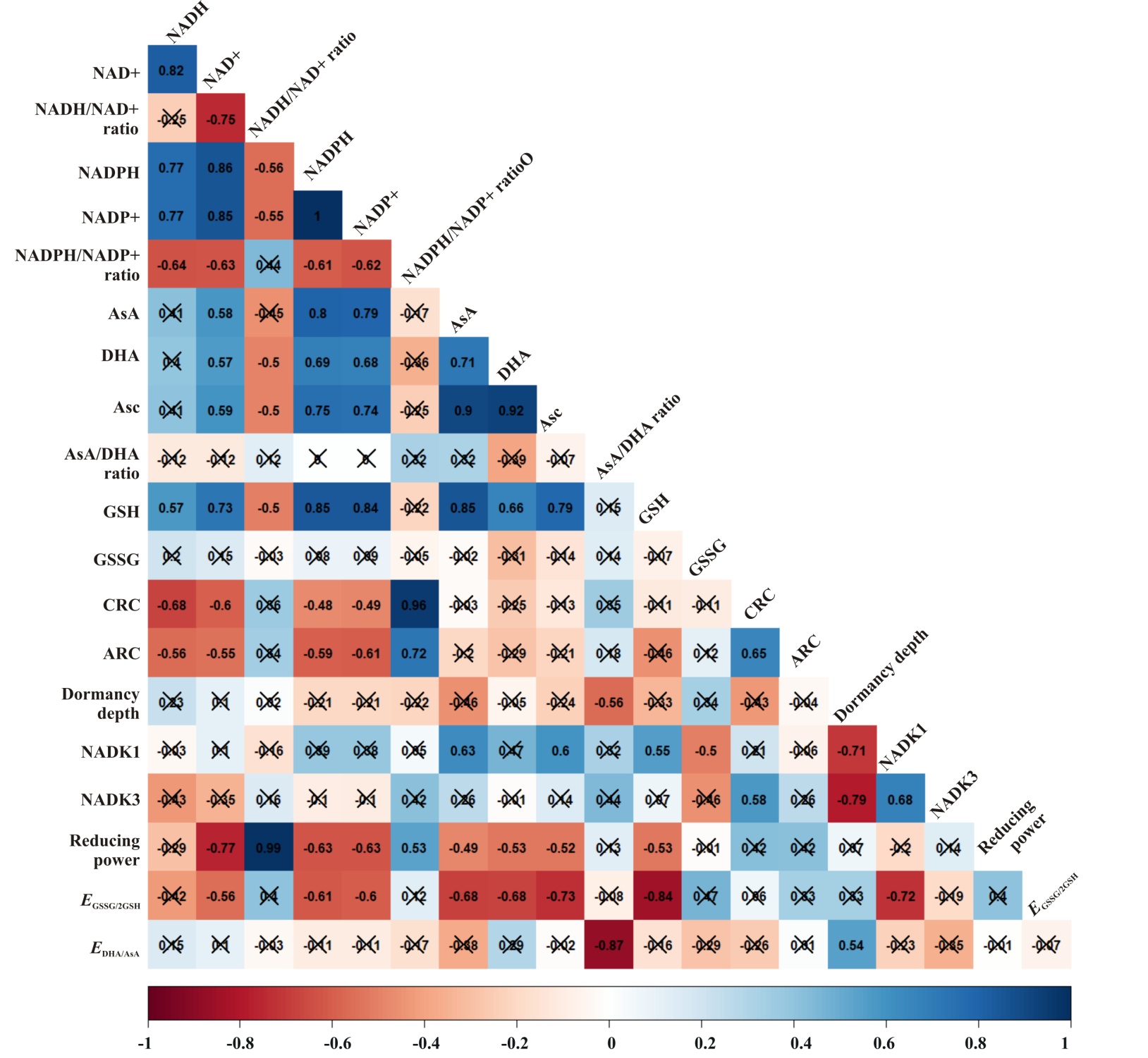
**

**B)** Norway maple cotyledons


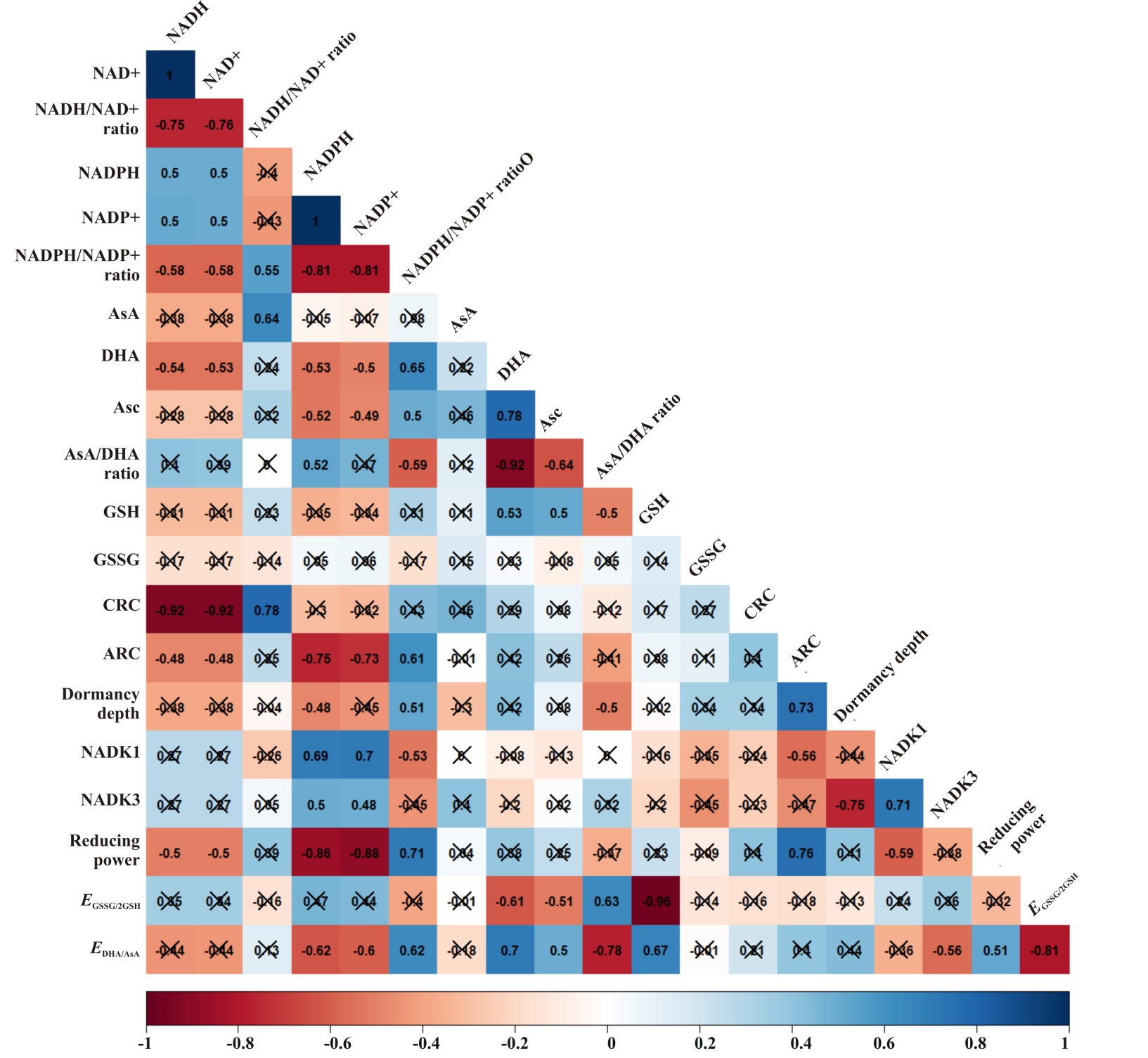


**C)** sycamore embryonic axes

**
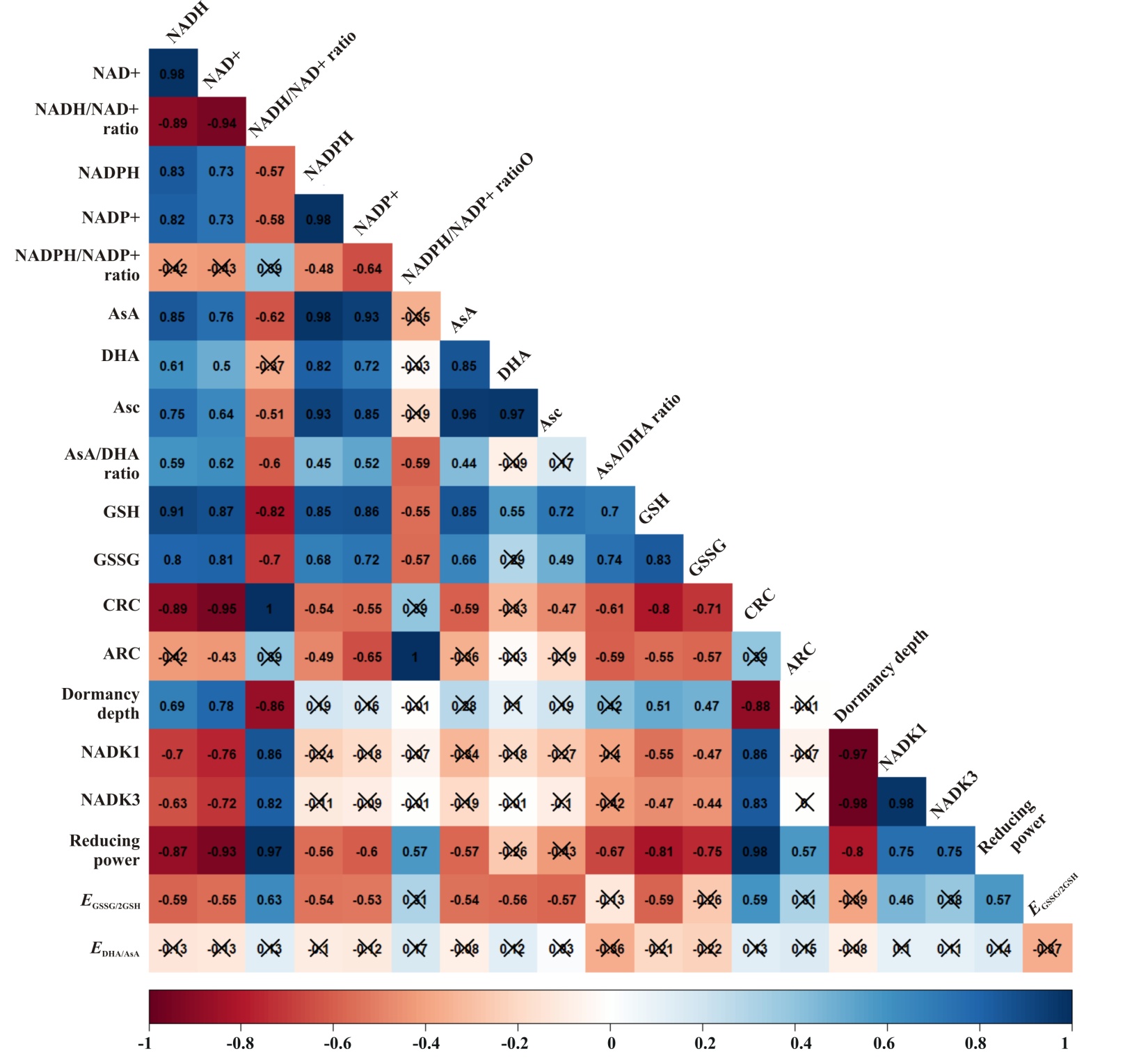
**

**D)** sycamore cotyledons

**
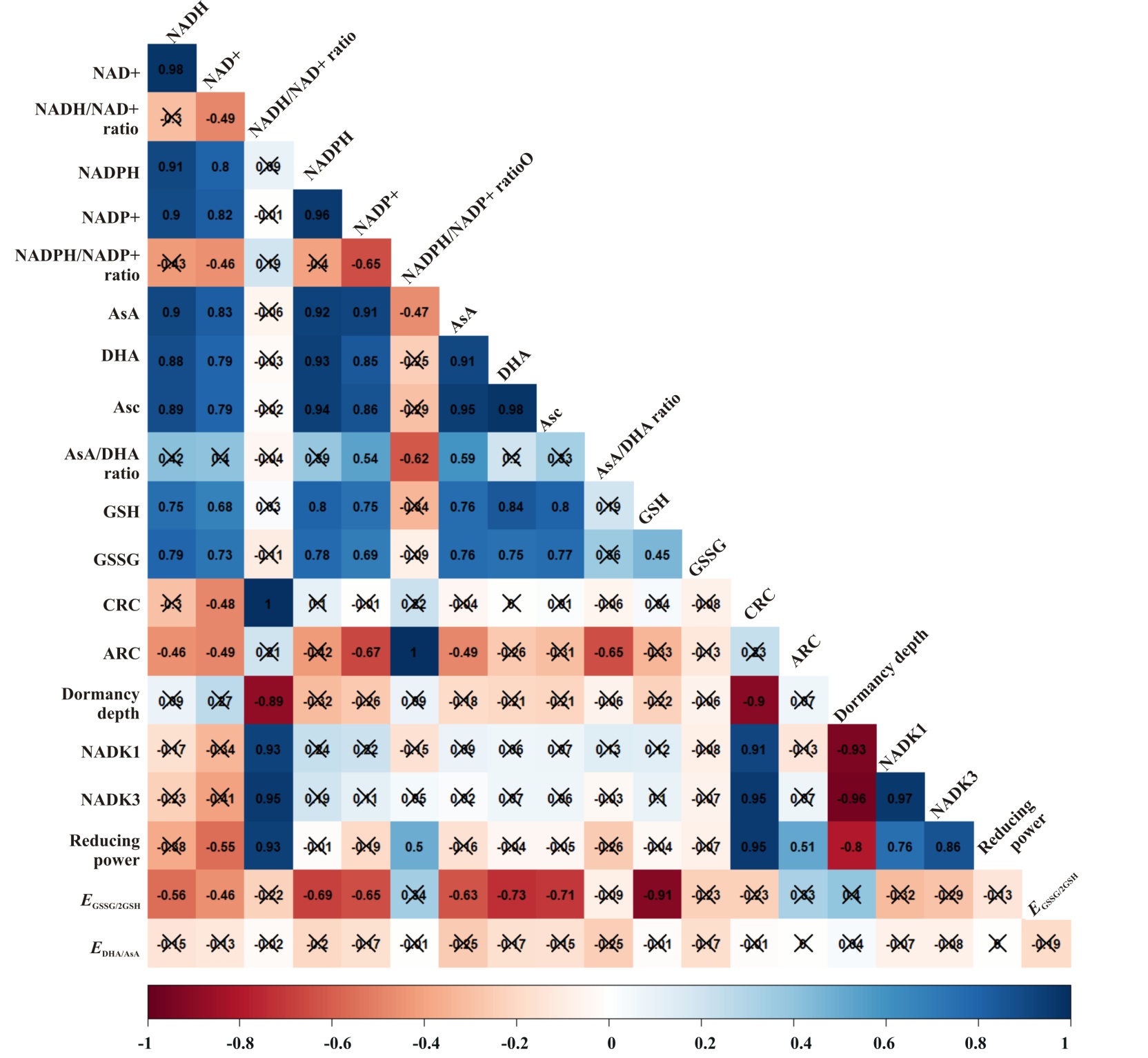
**
